# CBD-YOLO: a lightweight small-object detection algorithm for bud-stage Hangzhou white chrysanthemum in complex field environments

**DOI:** 10.3389/fpls.2026.1928425

**Published:** 2026-08-20

**Authors:** Yonghong Wu, Chennan Yu, Jianneng Chen, Xinyan Liu, Xinyao Wang

**Affiliations:** 1 School of Mechanical Engineering, Zhejiang Sci-Tech University, Hangzhou, China; 2 School of Mechanical Engineering, Tianjin University, Tianjin, China; 3 Zhejiang Zhigao Machinery Co., Ltd., Quzhou, China; 4 Zhejiang Key Laboratory of Intelligent Sensing and Robotics for Agriculture, Hangzhou, China

**Keywords:** bud-stage Hangzhou white chrysanthemum, lightweight model, precision agriculture, small-object detection, YOLOv9

## Abstract

Hangzhou white chrysanthemum is widely cultivated, and its bud stage has the highest commercial value. However, the optimal harvesting window is short, while manual harvesting is constrained by labor shortages and high labor costs. Accurate detection of bud-stage chrysanthemums is therefore essential for automated selective harvesting. This study proposes CBD-YOLO, a lightweight detector based on YOLOv9t, to address the small object size, dense distribution, and background interference of bud-stage chrysanthemums in field images. CBD-YOLO incorporates a normalized weighted fusion module at a high-level cross-scale fusion node to adaptively regulate the contributions of different feature branches, together with a lightweight contextual enhancement module to strengthen local feature representation in shallow high-resolution features. In experiments, CBD-YOLO achieved 85.85% precision, 78.80% recall, 89.88% mAP@0.5, and an F1-score of 82.08%, representing improvements of 3.29, 1.83, 2.13, and 2.62 percentage points over YOLOv9t, respectively. The model contained only 1.976 M parameters, required 7.60 GFLOPs, and had a model size of 4.70 MB. Embedded deployment validation on a Jetson NX platform showed that CBD-YOLO achieved 97.89 FPS with a mean model inference latency of 10.74 ms after TensorRT FP16 optimization. Ablation experiments, heatmap visualizations, and failure-case analysis further demonstrated the effectiveness and limitations of the proposed design. Overall, CBD-YOLO improves bud-stage chrysanthemum detection while maintaining a compact and deployment-friendly structure, providing a feasible technical basis for intelligent detection and automated selective harvesting under complex field conditions.

## 1 Introduction

Hangzhou white chrysanthemum is an important tea, food, and medicinal resource (Mei et al., 2023). Its cultivars and quality can be evaluated using chemical and omics-based approaches, and its active compounds are closely associated with functional properties such as antioxidant activity (Zhou et al., 2023). Tongxiang, Zhejiang Province, is the core production area of Hangzhou white chrysanthemum. The region has approximately 47,000 mu of planting area, accounting for about 60% of China’s white chrysanthemum cultivation area, and the output value of the whole industrial chain reaches about 1.6 billion yuan. Hangzhou white chrysanthemum is usually harvested from November to December. Among its growth stages, the bud stage has higher quality and market value, but the optimal harvesting period lasts only about 10 days. High labor costs and labor shortages have therefore become major constraints on industrial development (Zang et al., 2023). Accurate detection of bud-stage chrysanthemums is essential for automated selective harvesting and improved production efficiency.

Deep learning-based object detection has become a key technique in agricultural visual perception. It supports crop monitoring, growth-stage recognition, robotic harvesting, pest detection, and field management. Among existing detectors, the YOLO series has been widely adopted because it offers a favorable balance among detection accuracy, inference speed, and model size (Badgujar et al., 2024). Improved YOLO models have been applied to citrus fruit recognition (Ma et al., 2026), strawberry growth-state detection (Yang et al., 2025), crop pest monitoring (Wang N. et al., 2025), and tea-bud detection under complex backgrounds (Wang M. et al., 2024). To meet the deployment requirements of agricultural robots, unmanned aerial vehicles, and edge devices, recent studies have improved YOLO models through lightweight backbones, efficient convolutional modules, dynamic detection heads, reparameterized structures, and knowledge distillation (Lu et al., 2025; Feng et al., 2025).

Agricultural small-object detection places particular demands on feature representation and model efficiency. Common strategies include adding dedicated small-object detection heads, strengthening multi-scale feature fusion, incorporating attention or Transformer modules, and preserving spatial details in shallow features. Shi et al. (2024) integrated a Swin Transformer into YOLOv8 to enhance contextual representation for tea buds. Fang and Chen (2025) introduced a DSConv-based C2f module, Coordinate Attention, DySample, and SIoU loss for lightweight tea-bud detection. Zheng et al. (2024) proposed ESL-YOLO to improve small-object detection through feature enhancement, spatial-context-guided fusion, and local attention. These studies indicate that lightweight design, multi-scale feature fusion, and contextual enhancement are important directions for agricultural small-object detection.

Visual detection methods for Hangzhou white chrysanthemum and tea chrysanthemum have also evolved from traditional image processing to deep learning. Early studies mainly focused on target localization and flower segmentation (Yang et al., 2018, 2019). Later, YOLO-based models such as F-YOLO and TC-YOLO were developed for tea chrysanthemum detection under illumination variation, occlusion, flower overlap, and unstructured field environments (Qi et al., 2021, 2022). More recently, Hwc-YOLOv8n and improved YOLOv8s-based methods have been proposed for Hangzhou white chrysanthemum detection and flowering-stage classification (Yu et al., 2025; Shi et al., 2025).

Despite these advances, this task remains challenging. The targets are small and densely distributed, and they are morphologically similar to early-stage buds. They are also easily affected by unstructured backgrounds. Existing tea-bud detection models are difficult to transfer directly because tea buds and chrysanthemum buds differ in morphology, spatial distribution, and background interference. Current chrysanthemum detection models also struggle to balance robustness, growth-stage discrimination, and lightweight real-time deployment. A task-specific lightweight detector is therefore still needed for bud-stage chrysanthemum detection.

To address these challenges, this study adopts YOLOv9t as the baseline model and proposes CBD-YOLO for bud-stage chrysanthemum detection. A normalized weighted fusion module is introduced at a high-level cross-scale fusion node to adaptively balance the contributions of different input feature branches. In addition, a lightweight contextual enhancement module is used before shallow high-resolution features enter cross-scale fusion, strengthening local structural and contextual representations. These designs aim to improve the detection of small and densely distributed bud-stage chrysanthemums under complex field conditions while maintaining a compact model size and low computational cost.

## 2 Materials

### 2.1 Equipment and data collection

Image data were acquired using an Intel RealSense L515 solid-state LiDAR 3D depth camera. The device simultaneously records RGB and depth im-ages, with a maximum color-image resolution of 1920 x 1080 pixels and a depth-image resolution of 1280 x 720 pixels. Its depth measurement range is 0.25–9 m. During acquisition, the distance between the camera and the chrysanthemum canopy was maintained at approximately 50 cm.

To reduce the influence of strong field light and avoid overexposure in bud-stage chrysanthemum regions, manual exposure and fixed gain settings were used during acquisition. The camera exposure value was set to 39, and other parameters, including brightness and contrast, were maintained at medium levels. These settings preserved the color, texture, and morphological features of chrysanthemum buds and provided stable image inputs for model training.

All images were collected at the Hangzhou white chrysanthemum planting base of Yuanyuan Chrysanthemum Industry in Hangzhou, Zhejiang Province, China. The acquisition site is located at 120°24′ E and 30°37′ N. Image collection was conducted from 6 to 11 November 2025, during the suitable harvesting period for bud-stage chrysanthemum.

### 2.2 Data augmentation

**Figure 1:**
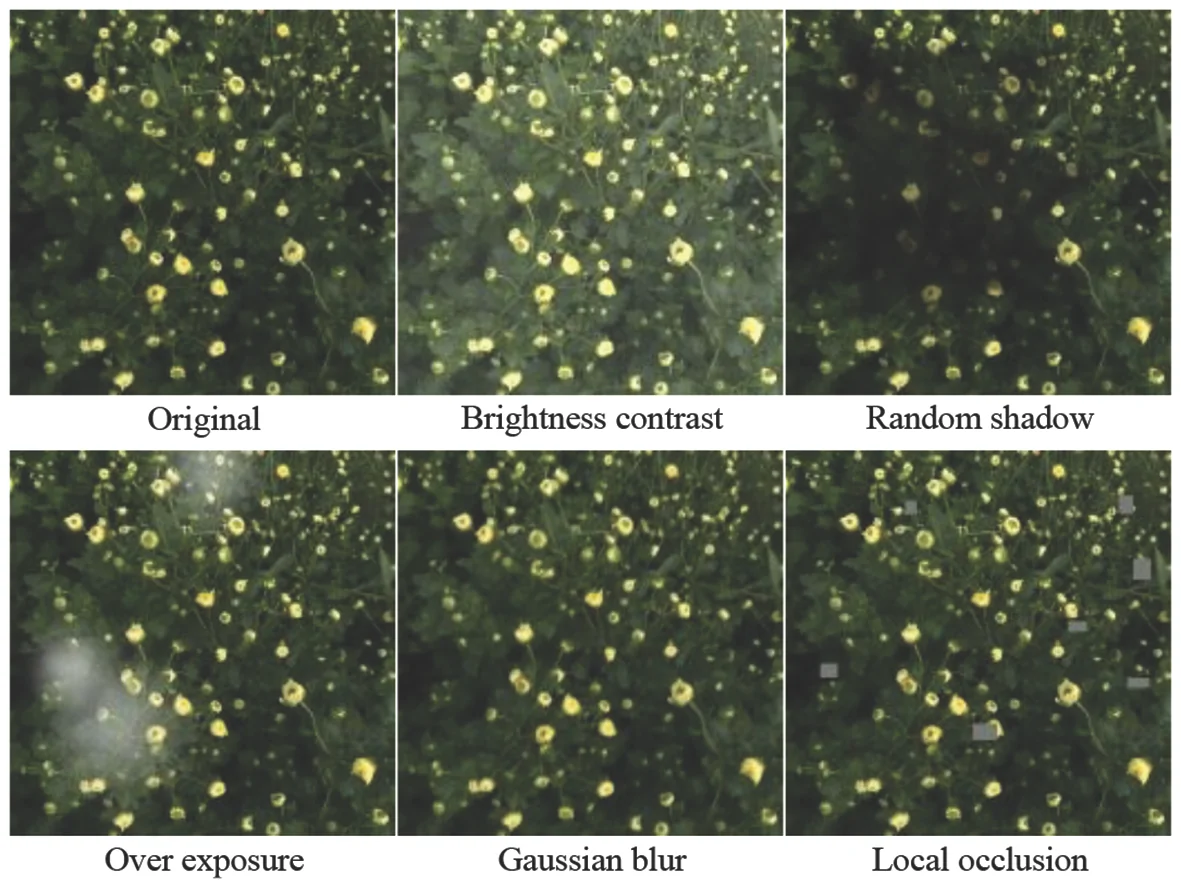
Data augmentation of the bud-stage chrysanthemum images.

With manually configured camera parameters, the acquired images showed stable quality, with no obvious overexposure or severe shadow interference. Under field conditions, however, illumination varies with weather, acquisition time, and camera angle, causing differences in brightness, contrast, and color distribution. Data augmentation was therefore applied to the original images to simulate complex field imaging conditions, enrich the training samples, and improve model robustness. The augmentation methods included brightness and contrast adjustment, random shadow, local overexposure, Gaussian blur and local occlusion. Examples of the augmented images are shown in **Figure 1**.

### 2.3 Dataset construction

**Figure 2:**
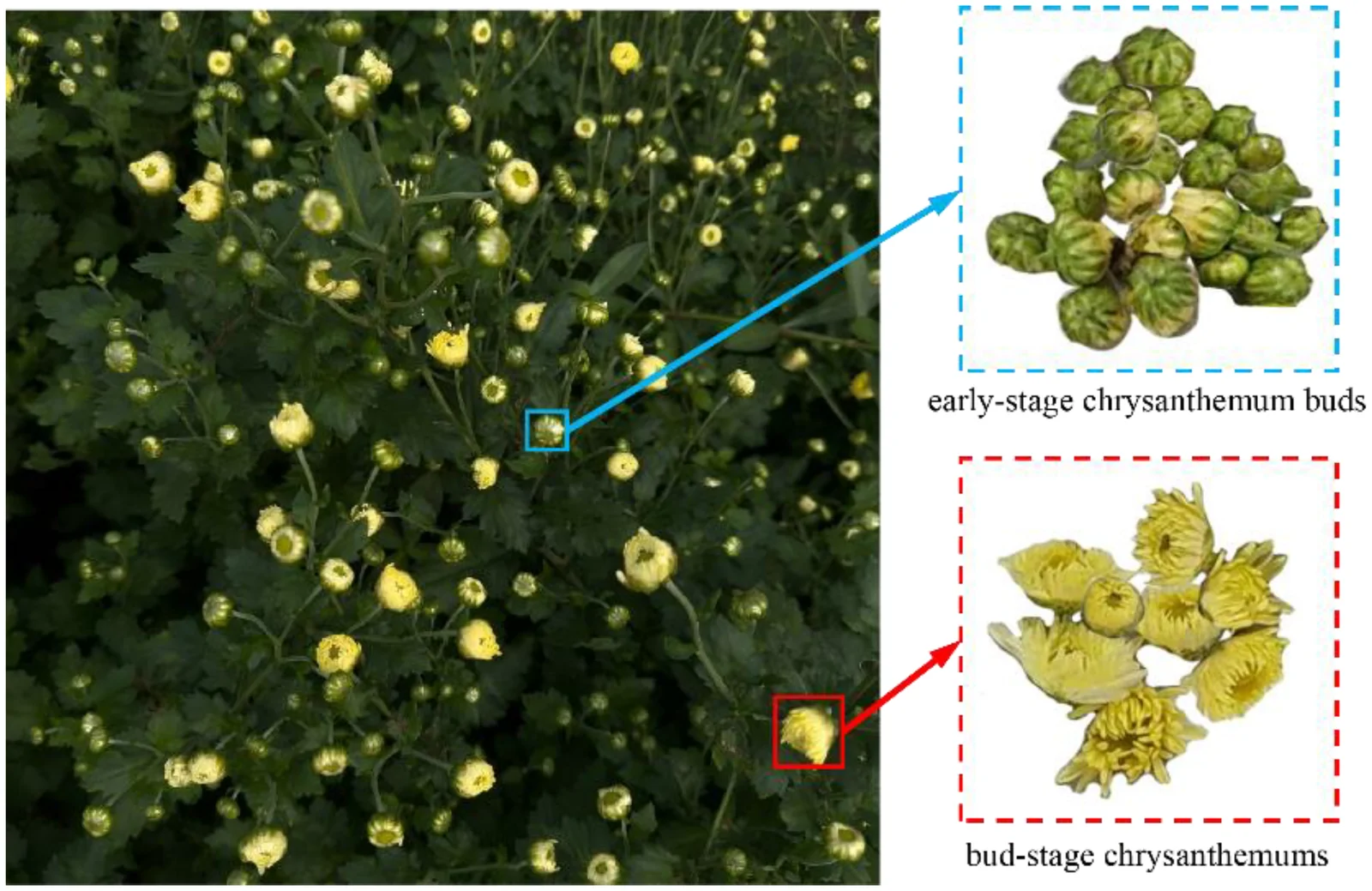
Feature comparison between bud-stage chrysanthemums and early-stage chrysanthemum buds.

During the harvesting period, some buds that are not harvested in time continue to develop into full-bloom chrysanthemums. In addition to numerous bud-stage chrysanthemums and early-stage buds, a small number of full-bloom chrysanthemums are therefore present in field images. Because full-bloom chrysanthemums differ markedly from bud-stage chrysanthemums in morphology, the risk of misclassifying them as bud-stage chrysanthemums is relatively low. To reduce annotation complexity and computational burden, this study annotated only bud-stage chrysanthemums and early-stage chrysanthemum buds, which have high visual similarity, as shown in **Figure 2**. This strategy enables the model to focus on subtle differences in morphology, scale and color between the two stages, thereby reducing inter-stage confusion and improving bud-stage chrysanthemums detection accuracy.

The positions and categories of bud-stage chrysanthemums and early-stage buds were annotated to convert the original field images into labeled training samples with explicit supervisory information. These annotations enabled the detection model to learn the morphological, color, scale, and spatial distribution features of Hangzhou white chrysanthemum at different growth stages. They also served as ground-truth references for model performance evaluation, including precision, recall, mAP, and F1-score. For densely distributed small-object detection in complex field environments, annotation quality directly affects small-object recognition and provides an important basis for improving model generalization and robustness.

In this study, Roboflow was used as the online annotation platform to label bud-stage chrysanthemums and early-stage chrysanthemum buds. The annotated images were divided into training, validation, and test sets at a ratio of 7:2:1. The numbers of bud-stage chrysanthemums in the training, validation, and test sets were 7991, 434, and 254, respectively, while the corresponding numbers of early-stage chrysanthemum buds were 24971, 1306, and 759. The annotation results were saved in YOLO format. Each annotation file contained the class index, the normalized x- and y-coordinates of the bounding-box center, and the normalized width and height of the bounding box.

## 3 Method

### 3.1 The architecture of the CBD-YOLO network

Bud-stage chrysanthemums are densely distributed targets that occupy only a small proportion of pixels in field images. Their limited pixel area, dense distribution, and strong background interference substantially increase the difficulty of detection. This challenge is mainly reflected in two aspects. First, multi-scale features contribute unequally to detection performance, and conventional feature fusion modules may introduce redundant background information during fusion, weakening the representation of key small-object features. Second, repeated downsampling can weaken or even remove edge, texture, and location information from small objects, thereby reducing the model’s localization and classification performance for densely distributed bud-stage chrysanthemums.

**Figure 3:**
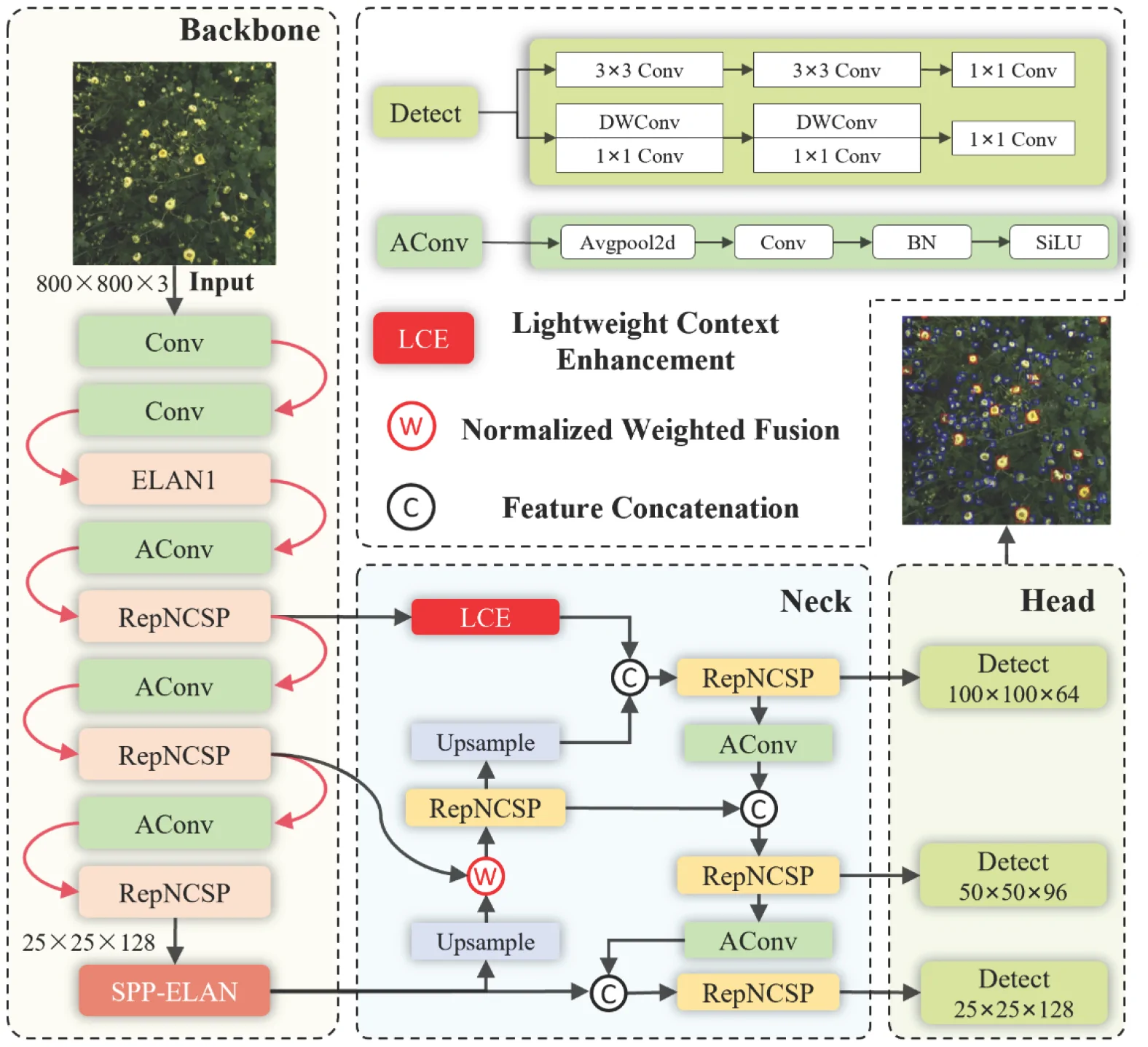
Schematic diagram of the CBD-YOLO architecture.

To address these problems, this study proposes CBD-YOLO, namely Chrysanthemum Bud Detection YOLO, a lightweight bud-stage chrysanthemum detection model based on YOLOv9t. The overall architecture is shown in **Figure 3**. CBD-YOLO integrates a normalized weighted fusion module (NWF) and a lightweight contextual enhancement module (LCE) into the YOLOv9t network. The NWF module assigns learnable weights to multi-level features during fusion, allowing the model to emphasize task-relevant feature layers and suppress invalid or redundant background interference. The LCE module enhances local texture representation and contextual awareness while maintaining low parameter and computational overhead. Together, these modules improve the recognition of densely distributed targets and enhance detection robustness in unstructured backgrounds.

#### 3.1.1 Overview of YOLOv9t

YOLOv9t maintains a small number of parameters and low computational complexity while achieving good detection accuracy and real-time performance. The network mainly consists of a backbone, a neck, and a detection head, with an adjustable input image size for different detection tasks. Considering the limited pixel area of the targets, the input image size was set to 800 × 800 to preserve more detailed information for small-object detection. The backbone of YOLOv9t adopts the GELAN structure to enhance feature extraction through hierarchical feature aggregation while maintaining a lightweight design. It mainly consists of Conv, ELAN1, AConv, RepNCSPELAN4, and SPPELAN modules. In the shallow layers, Conv and ELAN1 extract low-level visual features, such as textures and edges, whereas AConv performs feature downsampling and channel adjustment. RepNCSPELAN4 enhances feature representation through reparameterization and multi-branch feature aggregation, and SPPELAN expands the receptive field and integrates deep semantic information.

In the neck, YOLOv9t adopts the multi-scale feature fusion strategy commonly used in the YOLO series. Top-down and bottom-up feature propagation paths are used to enable cross-level feature fusion, allowing deep semantic information and shallow spatial details to jointly contribute to object representation. The detection head then performs object classification and bounding-box regression in parallel at three scales, P3/8, P4/16, and P5/32, to detect objects of different sizes.

Based on the performance comparison with mainstream object detection models, YOLOv9t achieves a favorable balance among model complexity, detection accuracy, and deployment efficiency, and was therefore selected as the baseline model in this study. Nevertheless, for these small and densely distributed targets in field scenes, the original YOLOv9t still has limitations in modeling local texture information of small objects and effectively fusing cross-scale features.

#### 3.1.2 Normalized weighted fusion module

**Figure 4:**
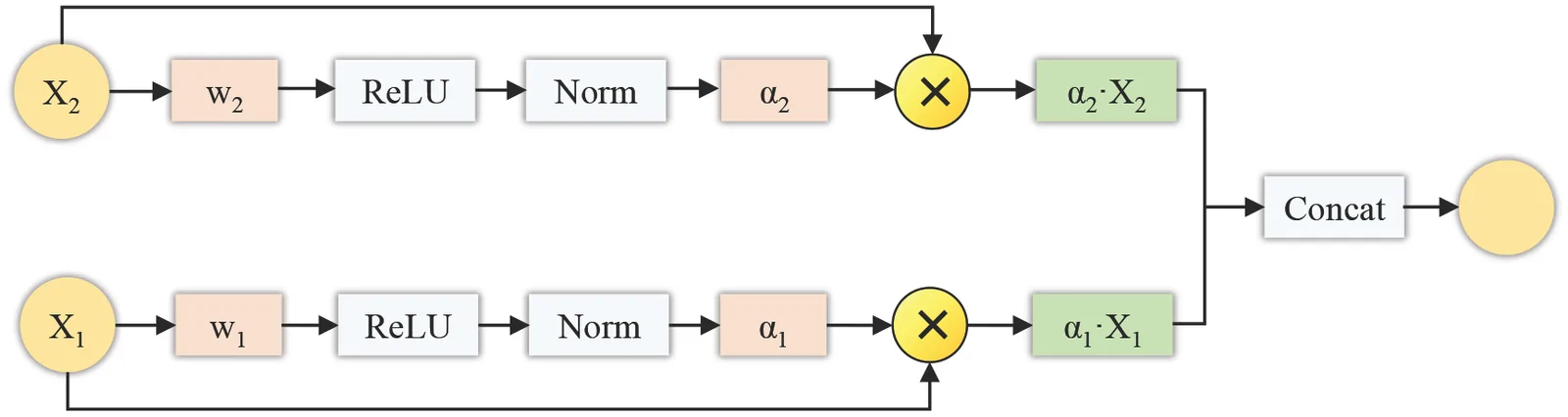
Structure of the normalized weighted fusion module.

In the original neck of YOLOv9t, multi-scale feature maps are fused mainly through direct channel-wise concatenation. This operation preserves the information from different input branches, but treats all branches equally and cannot adaptively regulate their relative contributions. For bud-stage chrysanthemum detection, the features involved in high-level cross-scale fusion may contain different amounts of useful semantic information because of the small target size, dense distribution, partial occlusion, and complex field backgrounds. Therefore, a lightweight normalized weighted concatenation module, referred to as NWF, was introduced at the selected high-level fusion node of the YOLOv9t neck, as shown in **Figure 4**.

Inspired by the normalized weighting strategy used in weighted feature fusion, NWF assigns a learnable scalar weight to each input branch before channel-wise concatenation. Unlike BiFPN, which generally performs normalized weighted summation within a repeatedly connected bidirectional feature pyramid, NWF does not reconstruct the overall neck architecture. Instead, it retains the original YOLOv9t neck topology and replaces only the selected direct-concatenation operation with normalized weighted concatenation. This design allows the relative contribution of each input branch to be adjusted while preserving its branch-specific channel information.

Let $X_{1}$ and $X_{2}$ denote the two input feature maps, and let ${\text{w}}_{1}$ and ${\text{w}}_{2}$ denote their corresponding learnable scalar weights. To ensure non-negative fusion coefficients, the weights are first constrained using the rectified linear unit and then normalized according to Equation 1:

(1) αi=ReLU(wi)∑j=12ReLU(wj)+ϵ,    i∈{1,2}

where ϵ is a small constant introduced to prevent division by zero. The normalized coefficients are then applied to the corresponding feature maps to the corresponding feature maps as expressed in Equation 2:

(2) $${\hat{X}}_{1}=\alpha_{1}X_{1},\;{\hat{X}}_{2}=\alpha_{2}X_{2}$$

Finally, the weighted feature maps are concatenated along the channel dimension as defined in Equation 3:

(3) $$Y=\text{Concat}({\hat{X}}_{1},{\hat{X}}_{2})$$

where *Y* denotes the fused output feature map.

Compared with direct concatenation, NWF adaptively adjusts the branch-level contribution before fusion while retaining the channel information of each input feature map. Compared with the weighted summation commonly used in BiFPN, weighted concatenation avoids directly collapsing different branches into the same feature space and remains compatible with the subsequent convolutional blocks of the original YOLOv9t neck. Moreover, NWF introduces only one learnable scalar for each input branch and several element-wise multiplication operations. Therefore, its additional parameter count and theoretical computational overhead are negligible.

In this study, NWF was applied only to the high-level cross-scale fusion stage, where semantic features are particularly important for distinguishing small chrysanthemum buds from complex backgrounds. By increasing the contribution of more informative feature branches and reducing that of less informative branches, NWF provides more discriminative fused features for the subsequent detection layers without substantially increasing model complexity.

#### 3.1.3 Lightweight contextual enhancement module

**Figure 5:**
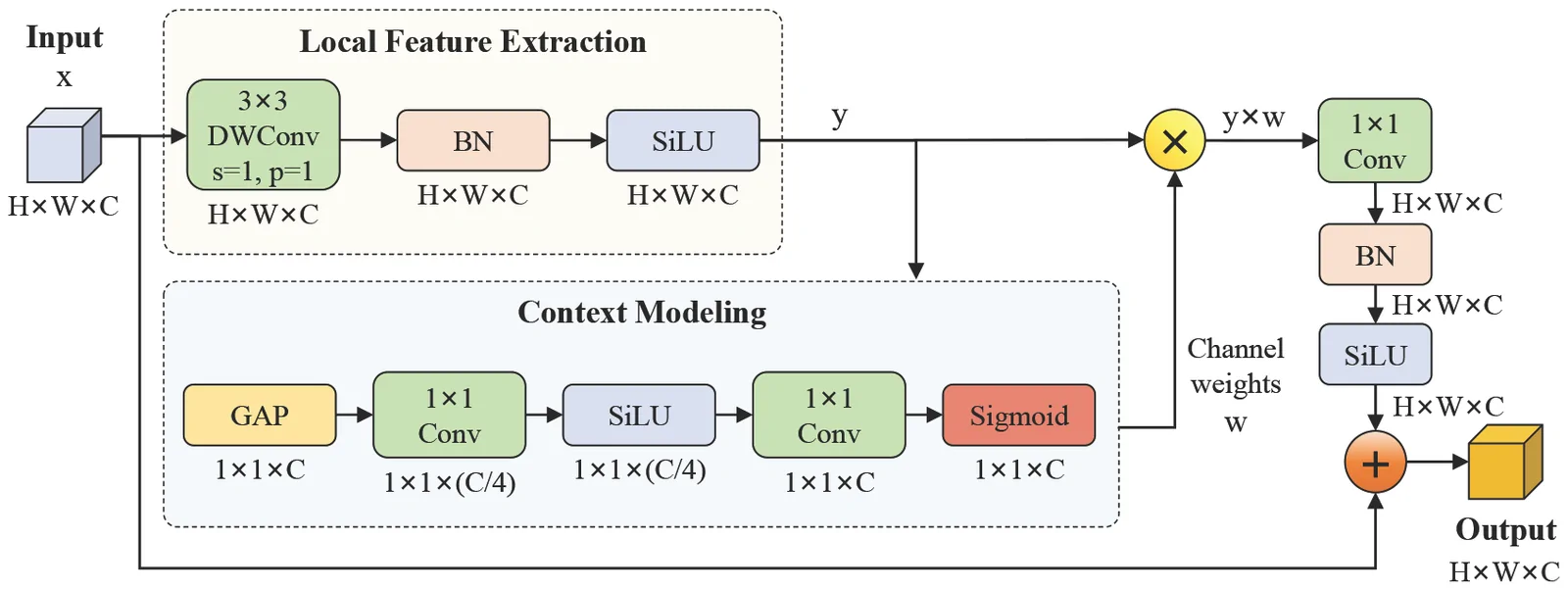
Structure of the lightweight contextual enhancement module.

In bud-stage chrysanthemum detection, shallow high-resolution features contain rich edge, texture, and spatial location information, which is essential for small-object localization. However, the original YOLOv9t does not sufficiently enhance shallow features and is susceptible to interference from leaves, branches, and illumination variation in complex field backgrounds. This limits the model’s ability to distinguish bud-stage chrysanthemums from background regions. To strengthen the representation of local details and contextual information in shallow features, an LCE module is introduced before the shallow high-resolution features are fed into cross-scale fusion, as shown in **Figure 5**.

The LCE module consists mainly of a local feature extraction branch, a context modelling branch, and a residual connection. Let *X* denote the input feature map. In the local branch, *X* is first processed by a depthwise convolution and then passed through batch normalization and the SiLU activation function. This operation extracts local texture, edge, and shape information with low computational complexity, and can be expressed as in Equation 4

(4) $$F_{l}=\delta \left(\text{BN}\left({\text{DWConv}}_{3\times 3}(X)\right)\right)$$

where $DWConv_{3\times 3}(\cdot )$ denotes a 3×3 depthwise convolution operation, BN(·) denotes batch normalization, δ(·) denotes the SiLU activation function, and $F_{l}$ denotes the output of the local feature extraction branch.

To enhance contextual representation, a context modelling branch is introduced. This branch takes the feature map $F_{l}$ output by the local feature extraction branch as input. Global average pooling is first applied to $F_{l}$ to obtain a global channel descriptor. Then, two 1×1 convolutions and a nonlinear activation function are used to model inter-channel dependencies, and a Sigmoid function is used to generate normalized channel weights. This process is formulated in Equation 5.

(5) $$F_{c}=Sigmoid\left({\text{Conv}}_{1\times 1}\left(\delta \left({\text{Conv}}_{1\times 1}\left(\text{GAP}(F_{l})\right)\right)\right)\right)$$

where GAP(·) denotes global average pooling, $Conv_{1\times 1}(\cdot )$ denotes a 1×1 convolution, and $F_{c}$ denotes the channel weights generated by the context modelling branch.

After obtaining the local feature $F_{l}$ and the context weights $F_{c}$, the module applies $F_{c}$ to the output of the local branch to recalibrate the local features channel by channel. This operation enhances the response to key target features while suppressing irrelevant background interference. The recalibration process is expressed in Equation 6.

(6) $$F_{r}=F_{l}⊗F_{c}$$

where ⊗ denotes channel-wise multiplication, and $F_{r}$ denotes the recalibrated feature map.

To further integrate the recalibrated feature information, $F_{r}$ is processed by a 1×1 convolution, followed by BN and SiLU activation to complete feature fusion. The fused feature is then added to the input feature *X* through a residual connection, yielding the final output feature *Y* of the LCE module, as formulated in Equation 7.

(7) $$Y=\delta \left(\text{BN}\left({\text{Conv}}_{1\times 1}(F_{r})\right)\right)+X$$

Through this design, the LCE module incorporates global contextual information with low computational overhead, enabling the adaptive enhancement of shallow features. Meanwhile, the residual connection preserves the original feature responses and reduces the loss of fine-grained details during feature enhancement. As shown in **Table 1**, unlike conventional attention mechanisms, LCE integrates local depthwise convolution, global channel recalibration, and residual feature preservation, thereby explicitly strengthening local spatial information in shallow high-resolution features. For bud-stage chrysanthemum detection under complex field conditions, this design improves the model’s recognition performance and detection robustness for small targets.

**Table 1:** Comparison of representative lightweight feature-enhancement methods.

Method	Structure	Computational complexity	Difference from LCE
SE Attention	Global pooling + FC-based channel attention	Low	Lacks explicit local spatial feature extraction.
ECA	Global pooling + 1D convolution-based channel attention	Very low	Models cross-channel interaction, but lacks spatial neighborhood modeling.
CBAM	Channel attention + spatial attention	Moderate	More complex.
Coordinate Attention	Directional pooling + coordinate attention	Moderate	Emphasizes directional long-range dependencies rather than local texture enhancement.
SimAM	Energy function-based parameter-free attention	Very low	Uses statistical importance estimation without learnable local feature extraction.
LCE	Local depthwise convolution + global channel recalibration + residual connection	Low	/

### 3.2 Evaluation metrics

To quantitatively evaluate model performance, this study used precision (P), recall (R), mean average precision (mAP), F1-score, number of parameters, and GFLOPs as the main evaluation metrics. Precision measures the correctness of model predictions, whereas recall measures the model’s ability to detect actual objects. For densely distributed target, higher recall indicates fewer missed detections, while higher precision indicates fewer false detections caused by background regions or visually similar objects. Precision and recall are calculated according to Equations 8 and 9, respectively.

(8) $$\text{P}=\frac{TP}{TP+FP}$$

(9) $$\text{R}=\frac{TP}{TP+FN}$$

where *TP* denotes the number of correctly detected objects, *FP* denotes the number of false detections, and *FN* denotes the number of real objects missed by the model.

To comprehensively evaluate detection performance under different confidence thresholds, this study further used mAP as an evaluation metric. Average precision (AP) represents the area under the precision–recall curve for a single category and is calculated according to Equation 10:

(10) $$AP=\int_{0}^{1}P(R)dR$$

where P(R) denotes precision as a function of recall. mAP denotes the mean AP across all categories and is calculated according to Equation 11:

(11) $$mAP=\frac{1}{N}\sum_{i=1}^{N}AP_{i}$$

where N denotes the total number of target categories, and $AP_{i}$ denotes the average precision of the i-th target category. The detection categories in this study include bud-stage chrysanthemum and early-stage chrysanthemum bud; therefore, N = 2. The mAP mentioned in this study refers to mAP@0.5, which represents the mean average precision when the intersection-over-union threshold is 0.5.

The F1-score jointly considers precision and recall, reflecting the balance between false detections and missed detections. It is calculated according to is calculated according to Equation 12:

(12) $$F1=\frac{2\times P\times R}{P\times R}$$

In addition, to evaluate the lightweight characteristics and deployment adaptability of the model, this study recorded the number of parameters and GFLOPs. The number of parameters reflects the model scale, whereas GFLOPs measure the computational complexity of forward inference. For practical applications such as intelligent harvesting, a smaller number of parameters and lower computational cost help improve deployment efficiency and real-time detection capability on edge devices.

## 4 Experiments

### 4.1 Experimental environment

As shown in **Table 2**, the neural network models were trained on a workstation equipped with an Intel Core i9-14900KF processor with 24 cores and 32 threads, 32 GB of system memory, and an NVIDIA GeForce RTX 4070 GPU with 12 GB of GPU memory. The software environment included Ubuntu Linux 24.04, CUDA 12.9, NVIDIA driver 580.126.09, PyTorch, and Python 3.8.20. In addition, embedded deployment validation was conducted on a Jetson NX platform, where the trained model was converted to a TensorRT FP16 engine for inference-speed evaluation.

**Table 2:** Experimental environment and training parameter settings.

Category	Item	Setting
Hardware	CPU	Intel Core i9-14900KF
	CPU cores / threads	24 cores / 32 threads
	System memory	32 GB
	GPU	NVIDIA GeForce RTX 4070
	GPU memory	12 GB
Software	Operating system	Ubuntu Linux 24.04
	CUDA version	CUDA 12.9
	NVIDIA driver	580.126.09
	Deep learning framework	PyTorch
	Python version	Python 3.8.20
Training parameters	Input image size	800 × 800
	Batch size	4
	Epochs	200

During training, the input image size was set to 800 × 800 to preserve fine-grained details of small bud-stage Hangzhou white chrysanthemum targets while keeping the computational cost manageable. Considering the input resolution, model complexity, and available GPU memory, the batch size and number of training epochs were set to 4 and 200, respectively, to ensure stable training and sufficient convergence. The optimizer was set to auto, with an initial learning rate of 0.01 and a final learning-rate factor of 0.01. The IoU threshold was set to 0.7, and the confidence threshold followed the default setting in Ultralytics. To reduce the effect of random initialization and data-order variation, the random seeds were set from 0 to 4, and each model was trained once under each seed. The reported detection results for all YOLO-based models are the mean values of the five independent training runs.

### 4.2 Comparison of different object detection algorithms

To verify the effectiveness of the proposed CBD-YOLO model, representative object detection algorithms were selected for comparison. The compared models included RT-DETR-L, RT-DETR-X (Zhao et al., 2024), Faster R-CNN (Ren et al., 2017), YOLOv8n (Jocher et al., 2023), YOLOv9t (Wang A et al., 2024), YOLOv10n (Wang C-Y et al., 2024), YOLOv11n (Khanam and Hussain, 2024), YOLOv12n (Tian et al., 2025), YOLOv26n (Jocher et al., 2026), and Hwc-YOLOv8n (Yu et al., 2025). Detection performance and model complexity were evaluated using precision, recall, F1-score, mAP@0.5, Params, GFLOPs, and model size. The results are shown in **Table 3**.

**Table 3:** Comparison of different models.

Models	P(%)	R(%)	F1-score(%)	mAP@0.5	Params(M)	GFLOPs(G)	Size (MB)
RT-DETR-l	82.40	84.00	83.20	82.50	31.987	103.40	66.3
RT-DETR-x	84.10	81.40	82.70	85.20	65.471	222.50	135.5
Faster R-CNN	87.50	64.52	74.30	61.70	41.300	133.95	165.7
YOLOv8n	84.81	75.03	79.54	87.56	3.006	8.10	6.30
YOLOv9t	82.56	76.97	79.46	87.75	1.971	7.60	4.70
YOLOv10n	79.53	75.07	77.18	81.62	2.266	6.50	5.80
YOLOv11n	82.31	78.40	80.12	87.40	2.583	6.30	5.50
YOLOv12n	83.05	76.50	79.58	87.38	2.557	6.30	5.60
YOLOv26n	81.44	73.72	77.37	82.88	2.375	5.20	5.40
Hwc-YOLOv8n	83.90	73.80	78.50	89.70	/	12.10	/
CBD-YOLO	85.85	78.80	82.08	89.88	1.976	7.60	4.7

As shown in **Table 3**, different detection models exhibited clear differences in bud-stage chrysanthemum detection. Faster R-CNN achieved the highest precision of 87.50%, indicating good discrimination for detected targets. However, its recall, F1-score, and mAP@0.5 were only 64.52%, 74.30%, and 61.70%, respectively, which were substantially lower than those of most YOLO-series models. In addition, Faster R-CNN required 41.300 M parameters, 133.95 GFLOPs, and 165.7 MB of storage, limiting its suitability for lightweight field deployment.

The Transformer-based RT-DETR models showed relatively strong recall and F1-score, but their computational costs were much higher. RT-DETR-L achieved a recall of 84.00% and an F1-score of 83.20%, while RT-DETR-X achieved a precision of 84.10% and an mAP@0.5 of 85.20%. However, RT-DETR-L required 31.987 M parameters and 103.40 GFLOPs, and RT-DETR-X required 65.471 M parameters and 222.50 GFLOPs. These results indicate that although RT-DETR models showed competitive detection capability, their high computational complexity and large model sizes may restrict their use in resource-constrained field platforms.

Among the YOLO-series models, YOLOv9t showed a favorable balance between detection accuracy and model complexity. It achieved precision, recall, F1-score, and mAP@0.5 values of 82.56%, 76.97%, 79.46%, and 87.75%, respectively, with only 1.971 M parameters, 7.60 GFLOPs, and a model size of 4.70 MB. Compared with YOLOv8n, YOLOv9t achieved a higher mAP@0.5 with fewer parameters and a smaller model size. Compared with YOLOv10n and YOLOv26n, YOLOv9t had slightly higher computational cost but achieved better mAP@0.5 and F1-score. Compared with YOLOv11n and YOLOv12n, YOLOv9t maintained comparable detection accuracy while requiring fewer parameters and a smaller model size. Considering detection performance, model scale, and lightweight deployment requirements, YOLOv9t was therefore selected as the baseline model in this study.

Compared with Hwc-YOLOv8n, a recently proposed model for Hangzhou white chrysanthemum detection, CBD-YOLO achieved better overall detection performance with lower computational cost. Hwc-YOLOv8n achieved 83.90% precision, 73.80% recall, 78.50% F1-score, and 89.70% mAP@0.5, with 12.10 GFLOPs. In contrast, CBD-YOLO achieved 85.85% precision, 78.80% recall, 82.08% F1-score, and 89.88% mAP@0.5, while requiring only 7.60 GFLOPs. Compared with Hwc-YOLOv8n, CBD-YOLO improved precision, recall, F1-score, and mAP@0.5 by 1.95, 5.00, 3.58, and 0.18 percentage points, respectively, while reducing computational cost by 4.50 GFLOPs.

Compared with the baseline YOLOv9t, CBD-YOLO improved all detection metrics while maintaining a compact model structure. Precision increased from 82.56% to 85.85%, recall increased from 76.97% to 78.80%, F1-score increased from 79.46% to 82.08%, and mAP@0.5 increased from 87.75% to 89.88%. The number of parameters increased only slightly from 1.971 M to 1.976 M, while GFLOPs remained unchanged at 7.60 G and the model size remained 4.70 MB. These results indicate that the proposed improvements enhanced detection accuracy without substantially increasing model complexity.

**Figure 6:**
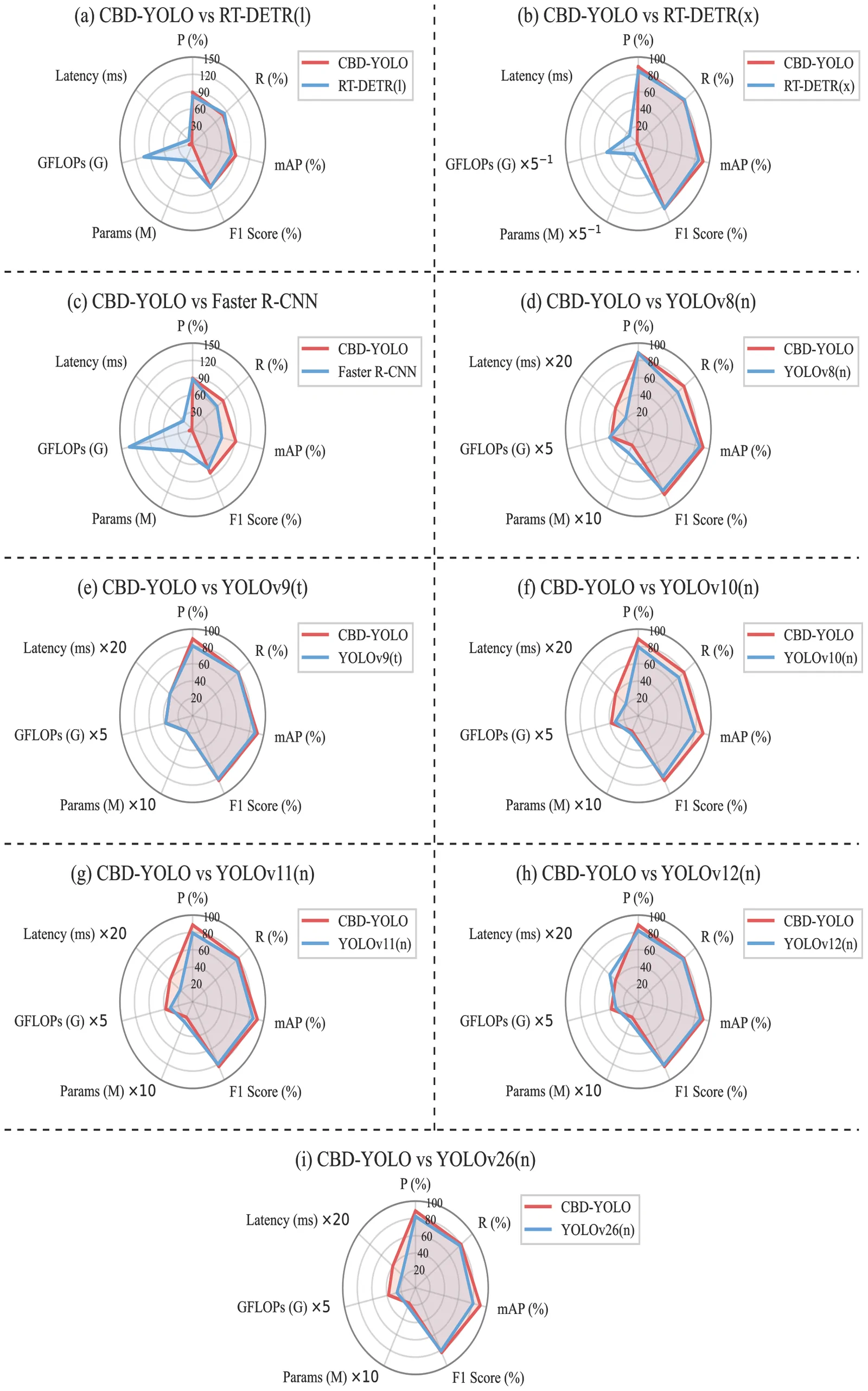
Performance comparison of CBD-YOLO with different object detection models based on radar charts: **(a)** CBD-YOLO vs. RT-DETR(l); **(b)** CBD-YOLO vs. RT-DETR(x); **(c)** CBD-YOLO vs. Faster R-CNN; **(d)** CBD-YOLO vs. YOLOv8(n); **(e)** CBD-YOLO vs. YOLOv9(t); **(f)** CBD-YOLO vs. YOLOv10(n); **(g)** CBD-YOLO vs. YOLOv11(n); **(h)** CBD-YOLO vs. YOLOv12(n); and **(i)** CBD-YOLO vs. YOLOv26(n).

**Figure 7:**
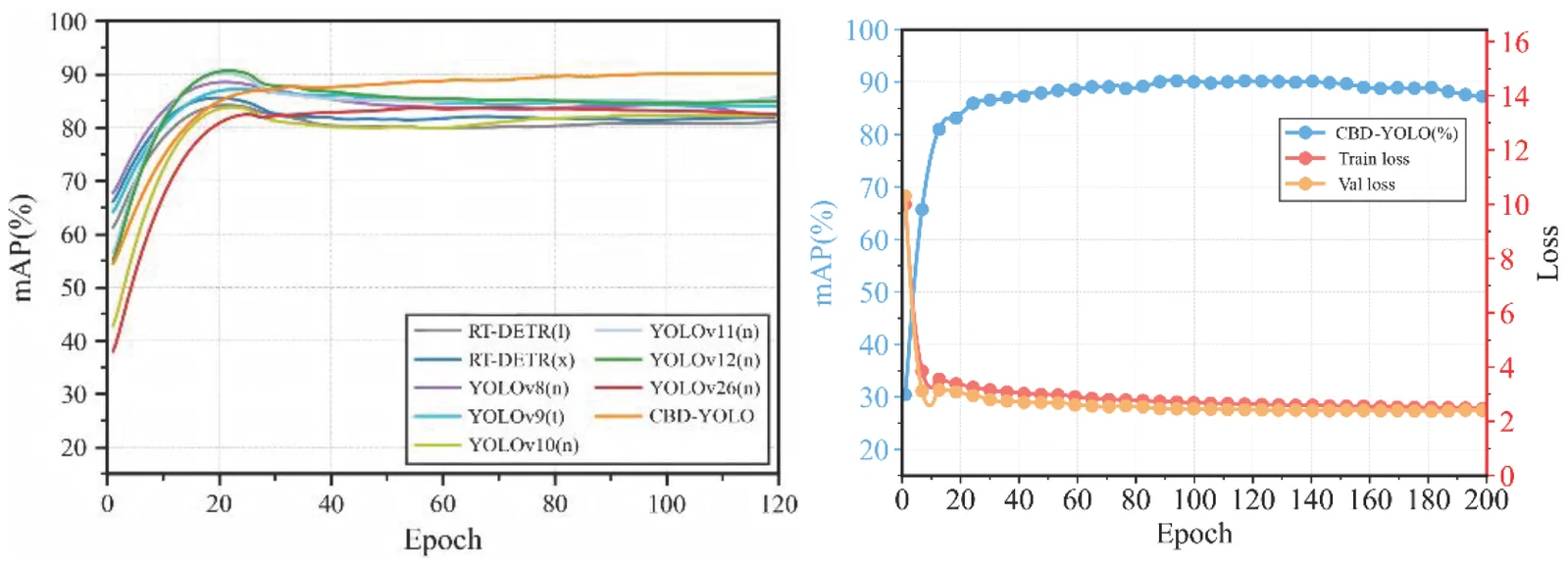
Evaluation of training performance and comprehensive performance of different detection models.

To further verify the reliability of the performance improvement, a statistical significance analysis was conducted between YOLOv9t and CBD-YOLO, as shown in **Table 4**. The results showed that the improvements in mAP@0.5 and F1-score were statistically significant, with p-values of 0.0410 and 0.0022, respectively. Although the improvements in precision and recall did not reach statistical significance, CBD-YOLO showed higher mean values for both metrics. These results indicate that CBD-YOLO achieved a more reliable improvement in overall detection performance, particularly in mAP@0.5 and F1-score. The radar chart in **Figure 6** summarizes the multi-metric comparison across models, and **Figure 7** presents the training behavior and final performance of the evaluated detectors.

**Table 4:** Statistical significance analysis of detection performance between YOLOv9t and CBD-YOLO.

Metric	YOLOv9t	CBD-YOLO	Mean difference	t	p	Significance
P (%)	82.56 ± 4.84	85.85 ± 1.14	+3.29	1.4795	0.2062	no
R (%)	76.97 ± 4.31	78.80 ± 2.76	+1.83	0.7995	0.4510	no
mAP@0.5 (%)	87.75 ± 1.64	89.88 ± 0.74	+2.13	2.6472	0.0410	yes
F1-score (%)	79.46 ± 0.61	82.08 ± 1.03	+2.62	4.8940	0.0022	yes

Overall, CBD-YOLO achieved the highest mAP@0.5 among all compared models and the best F1-score among the lightweight YOLO-based models. It maintained a compact structure with 1.976 M parameters, 7.60 GFLOPs, and a model size of 4.70 MB. These results demonstrate that CBD-YOLO provides a favorable trade-off between detection accuracy, model complexity, and deployment efficiency for this field detection task.

### 4.3 Ablation experiment

To verify the contribution of each improved module to model performance, ablation experiments were conducted using YOLOv9t as the baseline model. The effects of the NWF module, the LCE module, and their combination were evaluated separately. The evaluation metrics included precision (P), recall (R), F1-score, mAP@0.5, Params, and GFLOPs. The results are shown in **Table 5**.

**Table 5:** Results of the ablation experiment.

Models	P(%)	R(%)	F1-score(%)	mAP@0.5(%)	Params(M)	GFLOPs(G)
YOLOv9(t)	82.56	76.97	79.46	87.75	1.971	7.6
YOLOv9(t)+NWF	83.36	76.74	79.88	88.28	1.971	7.6
YOLOv9(t)+LCE	85.39	78.43	81.81	89.30	1.976	7.6
YOLOv9(t)+NWF+LCE	85.85	78.80	82.08	89.88	1.976	7.6

As shown in **Table 5**, the baseline YOLOv9t achieved precision, recall, F1-score, and mAP@0.5 values of 82.56%, 76.97%, 79.46%, and 87.75%, respectively, with 1.971 M parameters and 7.6 GFLOPs. After introducing the NWF module alone, precision increased to 83.36%, F1-score increased to 79.88%, and mAP@0.5 increased to 88.28%, whereas recall slightly decreased to 76.74%. These results indicate that NWF improved the discriminative ability of the model by enhancing multi-scale feature fusion and suppressing redundant background information. However, the slight decrease in recall suggests that weighted feature selection may also suppress some weak-response target features. In terms of model complexity, the number of parameters and GFLOPs remained unchanged, indicating that NWF introduced almost no additional computational cost.

After introducing the LCE module alone, the model achieved precision, recall, F1-score, and mAP@0.5 values of 85.39%, 78.43%, 81.81%, and 89.30%, respectively. Compared with the baseline YOLOv9t, these values increased by 2.83, 1.46, 2.35, and 1.55 percentage points, respectively. This result shows that LCE effectively enhanced local texture, edge, and contextual information in shallow high-resolution features, which improved the recognition of small bud-stage chrysanthemum targets in complex field backgrounds. The number of parameters increased only slightly from 1.971 M to 1.976 M, while GFLOPs remained 7.6 G, demonstrating the lightweight characteristic of the LCE module.

When NWF and LCE were jointly introduced into YOLOv9t, the resulting CBD-YOLO achieved the best overall performance. Its precision, recall, F1-score, and mAP@0.5 reached 85.85%, 78.80%, 82.08%, and 89.88%, respectively. Compared with the baseline model, these values increased by 3.29, 1.83, 2.62, and 2.13 percentage points, respectively. Compared with the model using only LCE, the combined model further improved precision, recall, F1-score, and mAP@0.5 by 0.46, 0.37, 0.27, and 0.58 percentage points, respectively. These results indicate that NWF and LCE had complementary effects. NWF strengthened adaptive feature fusion, whereas LCE enhanced local contextual representation for small targets. Their combination improved detection accuracy and stability while maintaining low model complexity.

To further determine the appropriate kernel size for the LCE module, a sensitivity analysis was conducted using different kernel sizes, as shown in **Table 6**. The results show that the 7 × 7 kernel achieved the best overall performance, with a recall of 78.80%, an F1-score of 82.08%, and an mAP@0.5 of 89.88%. Although the 9 × 9 kernel achieved the highest precision of 86.71%, its recall decreased to 75.79%, indicating that an excessively large kernel may weaken the model’s ability to detect some true targets. The 3 × 3 and 5 × 5 kernels achieved mAP@0.5 values of 89.53% and 89.25%, respectively, but their overall performance was lower than that of the 7 × 7 kernel. Therefore, the 7 × 7 kernel was selected for the LCE module because it provided a better balance among precision, recall, F1-score, and mAP@0.5.

**Table 6:** Sensitivity analysis of LCE kernel size on CBD-YOLO performance.

Model	Kernel size	P(%)	R(%)	F1-score(%)	mAP@0.5(%)
YOLOv9t	/	82.56	76.97	79.46	87.75
CBD-YOLO	3×3	85.84	77.50	81.42	89.53
CBD-YOLO	5×5	86.21	78.28	82.04	89.25
CBD-YOLO	7×7	85.85	78.80	82.08	89.88
CBD-YOLO	9×9	86.71	75.79	80.81	89.17

The effect of input resolution on detection performance was further analyzed, as shown in **Table 7**. For YOLOv9t, increasing the input resolution from 640 × 640 to 960 × 960 improved mAP@0.5 from 85.90% to 88.24%, indicating that higher-resolution inputs helped preserve more fine-grained features of bud-stage chrysanthemum targets. A similar trend was observed for CBD-YOLO, whose mAP@0.5 increased from 88.75% at 640 × 640 to 90.31% at 960 × 960. However, the 800 × 800 input resolution achieved a better balance among recall, F1-score, mAP@0.5, and deployment efficiency, with values of 78.80%, 82.08%, and 89.88% for recall, F1-score, and mAP@0.5, respectively. Therefore, 800 × 800 was adopted as the input resolution in the main experiments.

**Table 7:** Effect of input resolution on YOLOv9t and CBD-YOLO performance.

Method	Input resolution	P(%)	R(%)	F1-score(%)	mAP@0.5(%)
YOLOv9t	640×640	87.05	70.74	78.05	85.90
	800×800	82.56	76.97	79.46	87.75
	960×960	82.19	78.57	80.34	88.24
CBD-YOLO	640×640	87.02	78.78	82.70	88.75
	800×800	85.85	78.80	82.08	89.88
	960×960	89.33	73.32	80.54	90.31

In summary, the ablation results demonstrate that both NWF and LCE contributed positively to the detection performance of YOLOv9t. NWF improved feature fusion with almost no additional computational cost, while LCE substantially enhanced local contextual representation with only a slight increase in parameters. The joint introduction of NWF and LCE further improved the overall detection performance, confirming the effectiveness and complementarity of the two modules. The sensitivity analysis confirmed that a 7 × 7 kernel was suitable for the LCE module, and the input-resolution experiment showed that 800 × 800 provided a practical balance between detection accuracy and deployment efficiency. Therefore, the improved YOLOv9t structure embedded with both NWF and LCE, namely CBD-YOLO, was adopted as the final detection model in this study.

### 4.4 Heatmap visualization analysis

**Figure 8:**
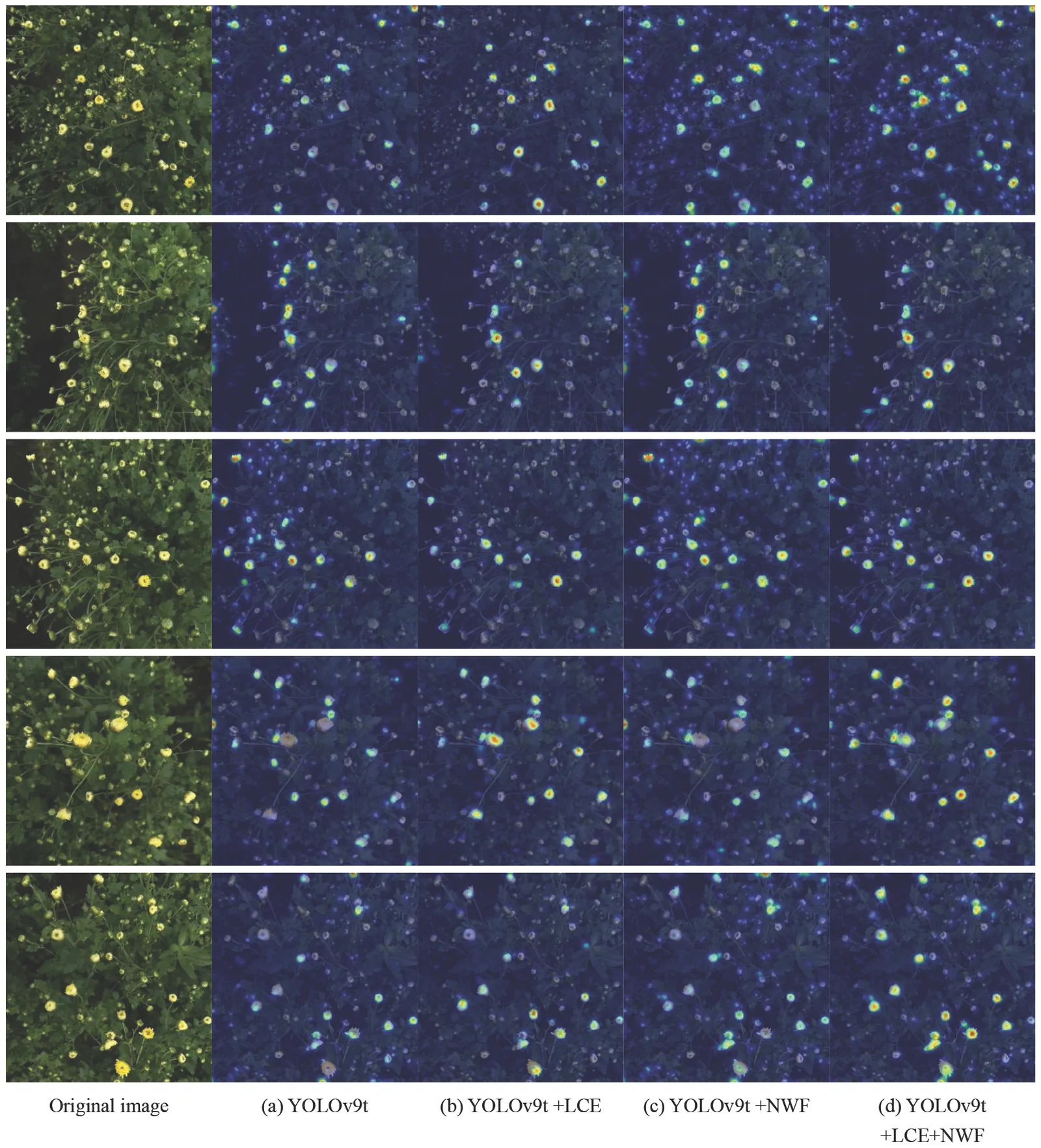
Heatmap comparison of attention regions generated by different improved components for the same images: **(a)** YOLOv9t; **(b)** YOLOv9t + LCE; **(c)** YOLOv9t + NWF; and **(d)** YOLOv9t + LCE + NWF.

To further analyze the effects of different improvement modules on the feature attention regions of the model, heatmap visualization was employed to compare YOLOv9t, the model with only the LCE module, the model with only the NWF module, and the model with both LCE and NWF modules. All models used the same input image, and the corresponding heatmap results are shown in **Figure 8**. The first column presents the original input image, whereas the remaining columns show the response distributions of different models over the target regions. In the heatmaps, the color gradually transitions from blue to green, yellow, and red, with colors closer to red indicating stronger responses of the model to the corresponding regions.

As shown in **Figure 8A**, the baseline YOLOv9t responded to some bud-stage chrysanthemum targets, but the high-response regions were relatively scattered, and several targets showed weak responses. This indicates that the baseline model had insufficient attention to densely distributed small objects in complex field backgrounds. After introducing LCE (**Figure 8B**), the model responses became more concentrated, and the weak-response target regions were enhanced, suggesting that LCE strengthened local texture, edge, and contextual information in shallow high-resolution features and improved the model’s attention to small objects and targets with blurred boundaries.

After introducing NWF (**Figure 8C**C), the model showed stronger focus on the main target regions, with more prominent high-response areas and reduced irrelevant background responses. Unlike LCE, which mainly enhances shallow fine-grained information, NWF improves adaptive feature selection during high-level cross-scale feature fusion, thereby increasing the contribution of effective semantic features and enhancing the discriminability of target-region responses.

When LCE and NWF were jointly introduced (**Figure 8D**), the model generated more concentrated, continuous, and stronger responses over bud-stage chrysanthemum regions, while background interference was further suppressed. These results indicate that LCE and NWF have complementary effects: LCE enhances shallow local context and fine-grained representation, whereas NWF improves the adaptive selection of high-level semantic features. Their combination enables the model to strengthen both local detail representation and semantic focus on key target regions, providing more discriminative feature inputs for the detection head.

Overall, the heatmap results further verify the effectiveness and rationality of the proposed improvement strategy in field scenes.

### 4.5 Deployment validation on Jetson NX

To further evaluate the deployment feasibility of CBD-YOLO on embedded platforms, the trained model was exported to ONNX format and further optimized into a TensorRT FP16 engine on a Jetson NX device. The model inference performance was evaluated using TensorRT trtexec over a 60 s test duration after 200 warm-up iterations. The results showed that, at an input size of 800 × 800, CBD-YOLO achieved 97.89 FPS on the Jetson NX platform, with a mean model inference latency of 10.74 ms. These results indicate that CBD-YOLO can be efficiently executed on embedded platforms and has practical application potential for lightweight field harvesting systems.

### 4.6 Analysis of failure cases

**Figure 9:**
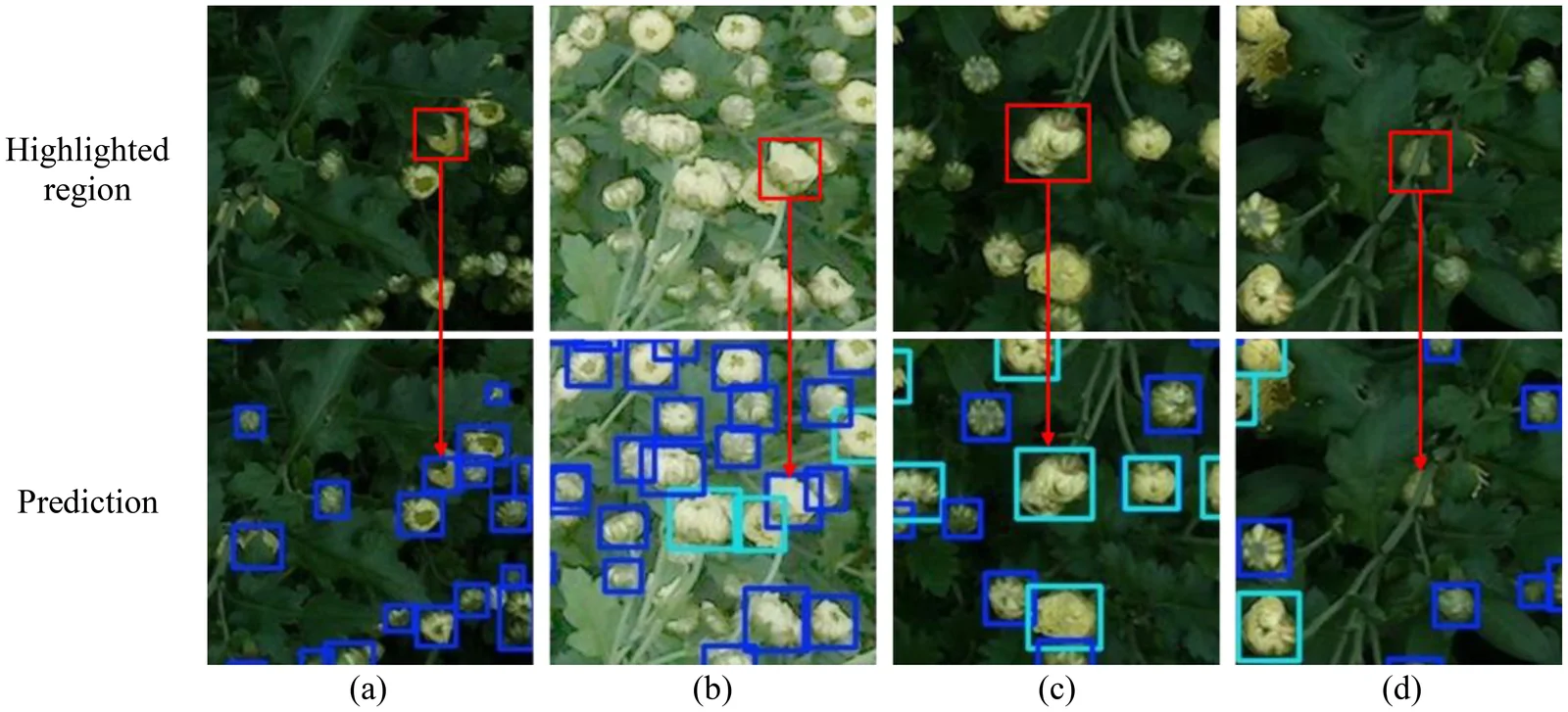
Representative failure cases of CBD-YOLO under complex field conditions: **(a)** false positive caused by occlusion; **(b)** false positive caused by overexposure; **(c)** missed detection caused by overlapping flowers; and **(d)** missed detection caused by severe occlusion. Blue boxes denote early-stage chrysanthemum buds, and cyan boxes denote bud-stage chrysanthemums.

To further analyze the detection performance of CBD-YOLO under complex field conditions, four representative failure cases were selected for visual analysis, as shown in **Figure 9**. The blue boxes denote early-stage chrysanthemum buds, whereas the cyan boxes denote bud-stage chrysanthemums. The results showed that the model errors mainly fell into two categories: misclassifying bud-stage chrysanthemums as early-stage chrysanthemum buds and failing to detect existing bud-stage chrysanthemum targets.

In **Figure 9A**, leaf occlusion caused part of the bud-stage chrysanthemum target to exhibit large green regions, leading the model to misclassify it as an early-stage chrysanthemum bud. This case indicates that, under partial occlusion, the discrimination of bud-stage chrysanthemums was susceptible to interference from background texture and color similarity. **Figure 9B** shows a false-positive case caused by overexposure. Strong illumination increased the local brightness of the image and weakened the color and texture differences between bud-stage chrysanthemums and early-stage chrysanthemum buds, thereby increasing the number of erroneous prediction boxes.

Missed detections mainly occurred in scenes where the target morphology was complex or the visible region was insufficient. As shown in **Figure 9(C)**, two bud-stage chrysanthemums overlapped with each other, resulting in indistinct target boundaries. In this case, the model tended to treat adjacent flowers as a single object, leading to missed detection of some targets. This result indicates that the instance separation capability of the model remained limited when targets of the same class were densely distributed and had highly overlapping boundaries. **Figure 9D** shows a missed-detection case caused by severe occlusion. Because only a small local region of the target was visible and the available visual features were insufficient, the model failed to extract stable shape and texture information and therefore did not generate a valid detection box.

Overall, the main failure sources of CBD-YOLO were associated with complex field factors, including extreme illumination changes, target overlap, and severe occlusion. These failure cases indicate that although the improved model achieved relatively stable bud-stage chrysanthemum detection in most scenarios, its robustness still needs to be further enhanced for low-visibility targets and images affected by extreme illumination.

## 5 Discussion and limitations

This study proposed CBD-YOLO, an improved YOLOv9t-based model for bud-stage chrysanthemum detection under complex field conditions. The main challenges of this task are that bud-stage chrysanthemum targets are small, densely distributed, and visually similar to surrounding leaves and adjacent flower stages. Under these conditions, detection performance depends not only on semantic feature representation but also on whether the model can preserve fine-grained local details and suppress redundant background information. To address these requirements, CBD-YOLO introduces the NWF and LCE modules into the YOLOv9t framework.

The NWF module assigns non-negative normalized learnable weights to different input branches before feature concatenation, allowing the model to adaptively adjust the contribution of each branch during high-level cross-scale feature fusion. By weighting the fusion process, NWF helps the network emphasize more discriminative features while reducing redundant responses caused by complex backgrounds. In contrast, LCE acts on shallow high-resolution features, where rich texture, edge, and local contextual information are still preserved. For bud-stage chrysanthemum detection, small targets can easily lose discriminative details during downsampling; therefore, enhancing shallow local features is particularly important. Thus, NWF mainly improves feature selection during cross-scale fusion, whereas LCE strengthens local representation for small targets.

The comparison with existing detectors further clarifies the practical value of the proposed design. CBD-YOLO improves detection accuracy through targeted feature refinement while maintaining a compact network structure, rather than by increasing model scale. This is particularly important for bud-stage chrysanthemum detection because field harvesting systems require both reliable recognition of small and densely distributed targets and efficient inference on resource-constrained devices. Therefore, the results indicate that, for this task, enhancing cross-scale feature fusion and shallow local contextual representation is more suitable for deployment than relying on larger detection backbones.

In field harvesting scenarios, the detector must process images rapidly while maintaining detection accuracy for small and densely distributed targets. The Jetson NX deployment validation showed that CBD-YOLO achieved 97.89 FPS with a mean model inference latency of 10.74 ms. Overall, these results indicate that CBD-YOLO has potential for embedded inference in lightweight field harvesting systems.

Several limitations remain in this study. First, although CBD-YOLO improved detection performance in most field scenes, the failure-case analysis showed that its robustness remains insufficient when target visibility is low or when field conditions deviate substantially from normal imaging conditions. Second, during data acquisition, the camera was manually operated and was not fixed on an actual harvesting platform. Therefore, the acquisition viewpoints and motion states may differ from those of a camera mounted on harvesting equipment, and the image acquisition setting cannot fully represent practical harvesting scenarios. These limitations indicate that CBD-YOLO still needs to be evaluated under more diverse and realistic field conditions before being integrated into a complete intelligent harvesting system.

## 6 Conclusions and future work

This study developed CBD-YOLO, a lightweight YOLOv9t-based model for detecting bud-stage chrysanthemums under complex field conditions. The model introduces a normalized weighted fusion module at a high-level cross-scale fusion node and embeds a lightweight contextual enhancement module in the shallow high-resolution feature branch. These two modules improve cross-scale feature fusion and local contextual representation while maintaining a compact network structure.

Compared with the baseline YOLOv9t, CBD-YOLO increased precision, recall, F1-score, and mAP@0.5 by 3.29, 1.83, 2.62, and 2.13 percentage points, respectively. The final model achieved a precision of 85.85%, a recall of 78.80%, an F1-score of 82.08%, and an mAP@0.5 of 89.88%, with 1.976 M parameters, 7.60 GFLOPs, and a model size of 4.70 MB. Statistical significance analysis further showed that the improvements in mAP@0.5 and F1-score were significant, indicating that CBD-YOLO achieved a more reliable improvement in overall detection performance. In addition, the embedded deployment validation showed that CBD-YOLO achieved 97.89 FPS with a mean model inference latency of 10.74 ms on the Jetson NX platform, demonstrating its potential for lightweight field deployment. The failure-case analysis indicated that although CBD-YOLO improved bud-stage chrysanthemum detection performance and attention to target regions, its robustness remained limited under extreme illumination, target overlap, and severe occlusion.

Overall, CBD-YOLO provides a lightweight and efficient detection approach for bud-stage chrysanthemum recognition in complex field environments. Future work will focus on optimizing the image acquisition system and integrating object detection with spatial localization and actuator control to support intelligent harvesting applications.

## Data Availability

The raw data supporting the conclusions of this article will be made available by the authors, without undue reservation.
